# Quantitative Relationship Between Solar Radiation and Grain Filling Parameters of Maize

**DOI:** 10.3389/fpls.2022.906060

**Published:** 2022-06-10

**Authors:** Yunshan Yang, Guangzhou Liu, Xiaoxia Guo, Wanmao Liu, Jun Xue, Bo Ming, Ruizhi Xie, Keru Wang, Peng Hou, Shaokun Li

**Affiliations:** ^1^The Key Laboratory of Oasis Eco-Agriculture, Xinjiang Production and Construction Corps/College of Agronomy, Shihezi University, Shihezi, China; ^2^Key Laboratory of Crop Physiology and Ecology, Ministry of Agriculture and Rural Affairs/Institute of Crop Sciences, Chinese Academy of Agricultural Sciences, Beijing, China; ^3^School of Agriculture, Ningxia University, Yinchuan, China

**Keywords:** maize, solar radiation, cultivar, grain filling rate, quantitative relationship

## Abstract

A quantitative understanding of the factors driving changes in grain filling is essential for effective prioritization of increasing maize yield. Grain filling is a significant stage in maize yield formation. Solar radiation is the energy source for grain filling, which is the ultimate driving factor for final grain weight and grain filling capacity that determine maize yield. Here, we first confirmed the quantitative relationships between grain filling parameters and photosynthetically active radiation (PAR) by conducting field experiments using different shading and plant density conditions and cultivars in 2019 and 2020 in Xinjiang, China. The results showed that with every 100 MJ m^−2^ increase in PAR, the average grain filling rate (*G*_ave_), maximum grain-filling rate (*G*_max_), and the kernel weight at the time of maximum grain-filling rate (*W*_max_) increased by 0.073 mg kernel^−1^ day^−1^, 0.23 mg kernel^−1^ day^−1^, and 0.24 mg kernel^−1^, and the time of maximum grain-filling rate (*T*_max_) delayed by 0.91 day. Relative changes in PAR were significantly and positively correlated with relative changes in yield and *G*_ave_. With every 1% change in PAR, yield and *G*_ave_ changed by 1.16 and 0.17%, respectively. From the perspective of grain filling capacity, DH618 was a more shade-resistant cultivar than XY335 and ZD958. It is urgent to breed maize cultivars with low light tolerance and high grain yield in the face of climate change, particularly the decrease in solar radiation.

## Introduction

Maize (*Zea mays* L.) is one of the most economically important crops, playing a vital role in maintaining food security in China and throughout the world (Hou et al., 2020; Liu et al., 2021c, 2022). Maize requires adequate solar radiation throughout the duration of growth to produce sufficient photoassimilates (Ren et al., 2016; Shi et al., 2018). However, recent studies have revealed that the intensity of photosynthetically active radiation (PAR) reaching the crop canopy is often reduced due to environmental pollution, overcast or rainy, and scant lighting the rainy weather frequently occurs during the crop growing season (Liu et al., 2012; Gao et al., 2017a; Zhang et al., 2019; Poonam et al., 2022). There have been varying degrees of reduction in radiation both nationally and worldwide (Ren et al., 2016; Zhou et al., 2020; Liu et al., 2021d), which could lead to reduced biomass production and maize yield (Hou et al., 2021). The reduction of light availability, especially during grain filling, threatens photosynthesis and carbohydrate synthesis, restricting maize yield (Jia et al., 2007; Zhang et al., 2007b; Ren et al., 2016; Gao et al., 2017a, 2020; Shao et al., 2020).

The grain filling stage is a crucial period during the entire growth season, with grain filling capacity determining final grain weight and yield (Shi et al., 2013; Cao et al., 2022). In the Southern and Huanghuaihai regions in China, maize yield has been limited by light deprivation during the grain filling stage (Wang et al., 2020a). The grain filling rate is one of the main limiting factors for yield increase (Ottaviano and Camussi, 1981; Xu et al., 2018). Zhou (2004) introduced that grain filling rate was one of the manifestations of grain’s ability to accumulate the photosynthate. Other studies have investigated both the rate and duration of grain filling, which jointly determine the formation of maize grain weight (Johnson and Tanner, 1972; Liu and Zhang, 2010). Grain filling in maize is influenced by various factors, for instance, genetics (Borrás et al., 2009; Li et al., 2020), irrigation methods and drought stress (Ahmad et al., 2022; Cao et al., 2022), fertilizer application (Zhang et al., 2021; Wu et al., 2022), and planting patterns and sowing densities (Jia et al., 2018). However, the amount of solar radiation received during the growing season is the most critical factor that determines the grain filling rate of maize (Shi et al., 2013, 2018; Chen et al., 2014; Rizzo et al., 2022).

Field shading is a common method used to vary solar radiation and study its effects on crop growth and development (Barnett and Pearce, 1983; Zhang et al., 2007b; Lu et al., 2013; Wang et al., 2020b). There have been many conclusions about the effects of shading on crop growth and the grain filling process in maize (Shi et al., 2013; Chen et al., 2014). It has been demonstrated that canopy shading reduces grain filling rate (Arisnabarreta and Miralles, 2008) and duration (Sandaña et al., 2009). Shi et al. (2013) reported that shading decreased the yield of summer maize by reducing the dry matter accumulation and maximum grain filling rate. Data published by Wang et al. (2020b) showed that shading stress at the grain filling stage depressed endosperm cell differentiation, which reduced storage capacity and decreased wheat yield. Furthermore, variations in solar radiation cause differences in grain filling characteristics at several growth stages (Shi et al., 2013; Chen et al., 2014). Previous studies have analyzed the qualitative effects of shading on grain filling in maize. However, the quantitative relationships between solar radiation and grain filling parameters have remained under-studied. In the present study, four shading levels were tested to investigate the effects of solar radiation on grain filling parameters, to deepen understanding of the process underlying grain weight formation, and develop effective interventions targeted to the critical stage of grain filling. We specifically aimed to (1) explore the response of final grain weight and grain-filling parameters to shading, and (2) quantify the relationship between grain filling parameters and PAR.

## Materials and Methods

### Experimental Design

Field experiments were conducted at the Qitai Farm in Xinjiang, Northwestern China (43°49′N, 89°48′E; 1,020 m above sea level) in the 2019 and 2020 growing seasons. This region is characterized by the most abundant sunshine hours in China (Xue et al., 2016). Meteorological data for the 2019 and 2020 maize growing seasons were obtained from a “Watch Dog” Data Logger (Spectrum Technologies, Inc., United States) located in the experimental field. The 2-year meteorological data are summarized in Table 1. The study was conducted using a split-split plot design with three replications. Three maize cultivars were used: Denghai618 (DH618), Xianyu 335 (XY335), and Zhengdan 958 (ZD958). These three cultivars have high yield potential and are widely planted throughout China (Hou et al., 2012; Gao et al., 2017b; Liu et al., 2021b). The sowing dates were April 19, 2019 and April 18, 2020. Two planting densities were tested: 7.5 × 10^4^ (D1) and 12 × 10^4^ plants ha^−1^ (D2). Four shading treatments were applied from the three-leaf stage until maturity: 15% (85% of natural light, 15% shaded; S1), 30% (70% of natural light, 30% shaded; S2), and 50% (50% of natural light, 50% shaded; S3) shading compared to natural light, and no shading (CK). The shade nets were built with temporary scaffolding and nylon nets, and a distance of 1.5 m was maintained between the top of the maize canopy and the shade nets to keep microclimate conditions (other than solar radiation) consistent with the unshaded portions of the field. Each experimental plot area was 11 × 10 m in an alternating narrow–wide-row planting pattern (0.4 + 0.7 m) and adjacent plots were spaced 1 m apart.

**Table 1:** Mean daily maximum temperature (*T*-max), minimum temperature (*T*-min), diurnal temperature variation (*T*d), solar radiation, relative humidity (RH), and precipitation (Pre) during the maize growing season at Qitai Farm, Xinjiang, China in 2019 and 2020.

Year	*T*-max (°C)	*T*-min (°C)	*T*d (°C)	Solar radiation (MJ m^−2^ day^−1^)	RH (%)	Pre (mm)
2019	25.9	11.5	18.7	9.8	52.6	138.5
2020	25.4	12.0	18.7	9.1	46.0	189.1

Maize was irrigated and fertilized using a drip irrigation system, with irrigation water pumped from groundwater (Zhang et al., 2017). In the 2-year field experiment, base fertilizers including urea (150 kg ha^−1^ N), super phosphate (225 kg ha^−1^ P_2_O_5_), and potassium sulfate (75 kg ha^−1^ K_2_O) were applied before sowing; additional urea (300 kg ha^−1^ N) was applied by fertigation during the entire irrigation period of the growing stage to ensure an adequate supply of nutrients. The experiments were conducted with no visible water stress, and pests and weeds were adequately controlled throughout the growing seasons.

### Sampling and Measurements

At the silking stage, plants at the same growth stage were labeled. From silking until maturity, five tagged ears from each plot were sampled at 10-day intervals; 100 grains were sampled from the middle part of the ear and oven-dried at 85°C to a constant weight. The grain-filling process of maize cultivars differing in maturity was analyzed using a logistic model:


$$y=A/\left(1+Be^{-\mathrm{Cx}}\right)$$

where *y* = grain weight (mg); *x* = number of days after silking; *A* is the potential kernel weight (mg), *B* and *C* are coefficients determined by regression. This model was used to evaluate the dynamics of accumulated grain weight in maize plants growth under different treatments (Gao et al., 2017b). The weight of a kernel at the time of the maximum grain-filling rate (*W*_max_) was equal to *A*/2 (Chen et al., 2014). The grain filling rate was derived from the first derivative of the sigmoidal equation. To determine yield (at 14% water content), an area of 16.5 m^−2^ comprising the central three rows of each plot (which were 5 m in length) were hand-harvested from each plot at maturity and grain moisture content was determined using a PM8188 portable moisture meter (Kett Electric Laboratory, Tokyo, Japan). PAR was measured in the wide and narrow rows with a diagonal orientation on clear days using a SunScan device (Delta-T Devices, Cambridge, United Kingdom; Liu et al., 2019; Yang et al., 2021a). Net photosynthetic rate (Pn) was measured under ambient conditions using a LI-6400 portable photosynthesis system (LI-COR Biosciences, Lincoln, Nebraska, United States) from the ear leaves of three representative plants during the grain-filling stage (30 days after silking). Cuvette conditions were 400 μmol CO_2_ mol^−1^, and PAR was 2,000 μmol m^−2^ s^−1^. The ambient temperature was 24°C–26°C. The cuvette area was 6 cm^2^.

### Statistical Analysis

Statistical analyses were performed in Excel 2016 (Microsoft, Redmond, WA, United States) and SPSS v18.0 (IBM SPSS, Chicago, IL, United States). The differences of yield, grain weight (*y*_P_), the average grain filling rate (*G*_ave_), the time of maximum grain-filling rate (*T*_max_), maximum grain-filling rate (*G*_max_), kernel weight at Tmax (*W*_max_), and *P*_n_ between different treatments were tested by using one-way ANOVA with the least significant difference test (LSD, *α* = 0.05). Pearson correlations were calculated to identify relationships between yield, PAR, *P*_n_, *y*_P_, and grain filling parameters. We conducted univariate analyses to examine interactions with *y*_P_, and grain filling parameters as dependent variables and the year (Y), cultivar (C), planting densities (D), and shading level (S) as independent variables. Figures were produced with Origin 2018 (OriginLab, Northampton, MA, United States).

## Results

### Yield and Phenological Information

Plant growth was recorded from sowing to maturity (Table 2). The three cultivars had a similar growth duration; DH618, XY335, and ZD958 had average growth durations (sowing to maturity) of 161.4, 165.9, and 162.7 days, respectively, in the 2 experimental years. For shading treatments, the silking and maturity stages delayed, and the duration of growth was longer than the control by 2.8, 3.1, and 7.6 days for S1, S2, and S3, respectively. The yield decreased in the order of S3 > S2 > S1 > CK after shading in 2019 and 2020, significantly. The average yield for DH618, XY335, and ZD958 under all shaded conditions decreased by 6.3, 7.2, and 5.2 t ha^−1^, respectively, at D1 and by 6.2, 7.5, and 7.3 t ha^−1^ at D2 compared to the CK yields. The grain yields of the three cultivars decreased in the order of XY335 > ZD958 > DH618. At lower planting density (D1), DH618 and XY335 were more sensitive to shading than ZD958 was, but yield decreases were smaller in DH618 than in XY335 and ZD958 at the higher planting density (D2). This indicated that DH618 were better able to tolerate low light under high-density planting.

**Table 2:** Yield, phenological information for the three maize cultivars at different shading levels (CK, natural light; S1, 15% of natural light; S2, 30% of natural light; and S3, 50% of natural light) and densities (D1 = 7.5 × 10^4^ plants ha^−1^ and D2 = 12 × 10^4^ plants ha^−1^) and the total intercepted PAR in the whole stage at the Qitai research station in 2019 and 2020.

Treatment	2019				2020			
	Silking date	Mature date	PAR MJ m^−2^	Yield (t ha^−1^)	Silking date	Mature date	PAR MJ m^−2^	Yield (t ha^−1^)
DH618-D1-CK	7/13	9/25	1097.1	20.39 a	6/28	9/23	1066.1	21.64 a
DH618-D1-S1	7/15	9/27	940.0	17.94 b	6/29	9/22	905.1	19.43 b
DH618-D1-S2	7/17	9/29	878.2	18.58 b	7/02	9/24	641.7	16.62 c
DH618-D1-S3	8/01	10/07	477.7	4.27 c	7/07	9/27	435.6	11.59 d
DH618-D2-CK	7/15	9/28	1162.0	20.05 a	7/03	9/22	1122.3	22.15 a
DH618-D2-S1	7/16	9/29	975.6	18.44 b	7/03	9/23	912.1	21.02 ab
DH618-D2-S2	7/18	9/26	890.6	18.38 b	7/03	9/22	691.7	18.96 b
DH618-D2-S3	8/02	10/07	470.3	0.58 c	7/09	9/29	439.3	12.03 c
XY335-D1-CK	7/15	9/29	1107.9	19.10 a	7/03	9/26	1023.9	22.30 a
XY335-D1-S1	7/16	10/05	942.3	15.03 b	7/04	9/27	837.4	20.58 b
XY335-D1-S2	7/17	10/04	856.3	15.64 b	7/05	10/01	609.8	15.82 c
XY335-D1-S3	8/01	10/07	380.8	3.40 c	7/10	10/01	409.4	10.77d
XY335-D2-CK	7/16	10/02	1231.1	21.57 a	7/03	9/24	1094.4	22.38 a
XY335-D2-S1	7/19	10/05	1038.5	19.25 b	7/06	9/30	953.2	22.26 a
XY335-D2-S2	7/19	10/05	944.0	15.87 c	7/08	9/30	730.6	16.49 b
XY335-D2-S3	8/5	10/07	466.1	3.02 d	7/12	10/05	516.0	10.25 c
ZD958-D1-CK	7/16	9/24	1150.0	18.44 a	7/02	9/25	1096.8	20.42 a
ZD958-D1-S1	7/18	9/29	940.3	18.13ab	7/02	9/25	912.6	19.08 a
ZD958-D1-S2	7/19	9/28	850.4	16.52 b	7/03	9/27	674.8	16.81 b
ZD958-D1-S3	8/06	10/05	374.7	2.80 c	7/09	9/27	465.5	11.78 c
ZD958-D2-CK	7/19	9/25	1219.1	18.62 a	7/03	9/24	1138.8	22.13 a
ZD958-D2-S1	7/19	10/02	990.5	15.83ab	7/05	9/26	989.2	20.19 a
ZD958-D2-S2	7/20	10/02	858.2	15.15 b	7/05	9/26	734.5	15.55 b
ZD958-D2-S3	8/10	10/05	419.9	1.04 c	7/14	10/01	443.1	10.75 c

Lowercase letters indicate significant differences between treatments at *p* < 0.05.

### Grain Weight and Grain Filling

As shown in Table 3, logistic fitting results showed that the potential grain weight *y*_P_ (when *x* = 80), differed between shading treatments; cultivar DH618 had the maximum *y*_P_ value and ZD958 had the minimum value. After shading, the potential grain weight *y*_P_ significantly decreased by 9.5% for ZD958, 6.7% for XY335, and 5.7% for DH618. The reduction in *y*_P_ at low planting density was higher than it was at high density (9.0 and 5.6%, respectively). Compared with CK, *y*_P_ decreased by 3.0, 6.7, and 12.1% in treatments S1, S2, and S3, respectively. *G*_ave_, *G*_max_, and *W*_max_ significantly decreased and *T*_max_ was delayed with increased shading levels (Table 3). *G*_ave_ (among all cultivars and densities) was 2.9, 6.5, and 11.7% lower in treatment groups S1, S2, and S3, respectively, than in the CK. Likewise, *G*_max_ was 4.5, 9.6, and 18.0% lower in S1, S2, and S3, respectively, compared to CK; *W*_max_ was 2.7, 5.9, and 9.1% lower than CK, and *T*_max_ was delayed by 1.0, 2.9, and 6.5 days, respectively. There were significant differences in grain filling parameters between cultivars and shading levels. On average across shading levels and densities, *G*_ave_ was 4.57, 4.23, and 4.18 mg kernel^−1^ day^−1^ for cultivars DH618, XY335, and ZD958; these values were 5.6, 6.4, and 9.1% lower than the CK, respectively. *G*_max_ was 9.24, 8.26, and 8.29 mg kernel^−1^ day^−1^, representing a decrease of 9.6, 10.4, and 12.1%, respectively, compared to the CK. *T*_max_ was 37.9, 41.4, and 41.7 days for cultivars DH618, XY335, and ZD958, respectively, which were later than the CK by 2.7, 4.0, and 3.8 days. *W*_max_ was 192.7, 180, and 177.5 mg kernel^−1^ day^−1^ for the three cultivars, a decrease of 4.9, 5.1, and 7.7% compared to the CK. Shading treatment had a great effect on *G*_ave_ and *W*_max_ at low planting density (D1), whereas high planting density (D2) had a great effect on *G*_max_ and *T*_max_ compared with CK on average across all shading levels and cultivars.

**Table 3:** Characteristic parameters of maize at grain-filling stage under different treatments.

Treatment	A	B	C	*y*_p_ (mg kernel^−1^)	*G*_ave_ (mg kernel^−1^ day^−1^)	*T*_max_ (d)	*G*_max_ (mg kernel^−1^ day^−1^)	*W*_max_ (mg kernel^−1^)
DH618-D1-CK	415.8 ± 10 a	31.7 ± 0 a	0.1	410.5 ± 22.1 a	4.93 ± 0.08 a	35.3 ± 0 b	10.1 ± 0.2 a	207.9 ± 4.1a
DH618-D1-S1	406.5 ± 10.7 ab	34.8 ± 8.8 a	0.1	401.5 ± 7.7 a	4.84 ± 0.02 a	35.5 ± 0.5 b	10.1 ± 0.2 a	203.3 ± 4.4 ab
DH618-D1-S2	388.1 ± 10 b	44.8 ± 11.8 a	0.1	383.2 ± 15.8 a	4.65 ± 0.01 ab	37.6 ± 1 ab	9.9 ± 0.2 a	194.1 ± 4.1 b
DH618-D1-S3	361.6 ± 16.2 c	45.5 ± 18 a	0.1	354.1 ± 3.3 b	4.3 ± 0.27 b	40 ± 1.6 a	8.7 ± 0.3 b	180.8 ± 6.6 c
DH618-D2-CK	384.7 ± 12.1 a	43.5 ± 14.1 a	0.1	380.1 ± 4.3 a	4.61 ± 0.1 a	36.6 ± 1 b	9.8 ± 0.2 a	192.4 ± 4.9 a
DH618-D2-S1	381.5 ± 12.9 a	37.8 ± 11.7 a	0.1	375.4 ± 5.2 a	4.54 ± 0.02 a	37.2 ± 0.9 b	9.3 ± 0.2 a	190.8 ± 5.3 a
DH618-D2-S2	380.2 ± 15.8 a	31.8 ± 9.2 a	0.09	368.9 ± 8.8 a	4.44 ± 0.02 a	40 ± 0.4 a	8.3 ± 0.3 b	190.1 ± 6.5 a
DH618-D2-S3	364.4 ± 16.7 a	35 ± 10.9 a	0.09	352.1 ± 4.4 b	4.25 ± 0.03 b	41.5 ± 0.5 a	7.9 ± 0.3 b	182.2 ± 6.8 a
XY335-D1-CK	387 ± 14.4 a	34.9 ± 10.3 a	0.09	378.4 ± 27.1 a	4.57 ± 0.18 a	38.4 ± 0.4 b	8.9 ± 0.2 a	193.5 ± 5.9 a
XY335-D1-S1	375.5 ± 15 ab	35.4 ± 10.8 a	0.09	366 ± 3 a	4.42 ± 0.1 a	39.8 ± 0.5 ab	8.5 ± 0.2 a	187.8 ± 6.1 ab
XY335-D1-S2	353.6 ± 15.7 bc	44.2 ± 15.4 a	0.09	343.8 ± 21.7 ab	4.17 ± 0.08 a	41.6 ± 0.9 a	8.1 ± 0.3 a	176.8 ± 6.4 bc
XY335-D1-S3	336.7 ± 15.1 c	46.7 ± 17.5 a	0.09	328.5 ± 6.6 b	3.99 ± 0.03 b	41.2 ± 0.8 a	7.1 ± 0.3 b	168.4 ± 6.2 c
XY335-D2-CK	362.3 ± 10.8 a	45.2 ± 13.2 a	0.1	357 ± 7.4 a	4.33 ± 0.01 a	38.4 ± 0.4 c	9.1 ± 0.2 a	181.2 ± 4.4 a
XY335-D2-S1	360.3 ± 12.3 a	44.1 ± 12.7 a	0.09	352.2 ± 4.2 a	4.27 ± 0.02 a	40 ± 0.8 bc	8.5 ± 0.2 ab	180.2 ± 5 a
XY335-D2-S2	346.3 ± 13.6 a	48.5 ± 15 a	0.09	336.2 ± 4.5 b	4.09 ± 0.03 b	42.4 ± 0.9 b	8 ± 0.2 b	173.2 ± 5.6 a
XY335-D2-S3	358.2 ± 22.3 a	52.3 ± 16 a	0.08	329.9 ± 6.4 b	4.02 ± 0.1 b	49.6 ± 0.9 a	7.2 ± 0.2 c	179.1 ± 9.1 a
ZD958-D1-CK	399.6 ± 13 a	38.4 ± 11 a	0.1	392.5 ± 7.8 a	4.75 ± 0.01 a	38.4 ± 0.7 b	9.6 ± 0.1 a	199.8 ± 5.3 a
ZD958-D1-S1	378 ± 13.4 ab	39.5 ± 12 a	0.09	370.6 ± 6.6 b	4.48 ± 0.02 b	39.1 ± 1.3 b	9 ± 0.1 b	189 ± 5.5 ab
ZD958-D1-S2	367.5 ± 14.5 b	45.5 ± 14.9 a	0.09	358.6 ± 3.2 b	4.35 ± 0.06 b	40.4 ± 0.9 ab	8.6 ± 0.2 b	183.8 ± 5.9 b
ZD958-D1-S3	329.2 ± 18 c	58.6 ± 32.4 a	0.1	320.7 ± 11.6 c	3.91 ± 0.04 c	42.6 ± 0.9 a	7.9 ± 0.2 c	164.6 ± 7.3 c
ZD958-D2-CK	355.1 ± 10.8 a	45 ± 12.6 a	0.1	348.7 ± 22.5 a	4.23 ± 0.01 a	39.3 ± 1.9 b	8.7 ± 0.2 a	177.6 ± 4.4 a
ZD958-D2-S1	340.2 ± 11.5 a	53.6 ± 16.6 a	0.1	333 ± 17.7 a	4.06 ± 0.04 b	41.1 ± 2.1 b	8.3 ± 0.2 a	170.1 ± 4.7 a
ZD958-D2-S2	331.9 ± 12.4 a	51.2 ± 16 a	0.09	323.3 ± 9.5 a	3.93 ± 0.05 b	42 ± 2.2 b	7.8 ± 0.2 a	166 ± 5.1 a
ZD958-D2-S3	339.1 ± 28.8 a	48.1 ± 19.9 a	0.08	305.1 ± 1.4 b	3.71 ± 0.04 c	50.8 ± 3 a	6.4 ± 0.5 b	169.6 ± 11.8 a

A, B, and C were the coefficients of the Logistic equation; *y*_p_, potential grain weight (*x* = 80). *G*_ave_, average grain filling rate; *T*_max_, time to maximum grain filling rate; *G*_max_, maximum grain-filling rate; and *W*_max_, kernel weight at *T*_max_. Lowercase letters indicate significant differences between treatments at *p* < 0.05.

### Photosynthetic Capacity

**Figure 1:**
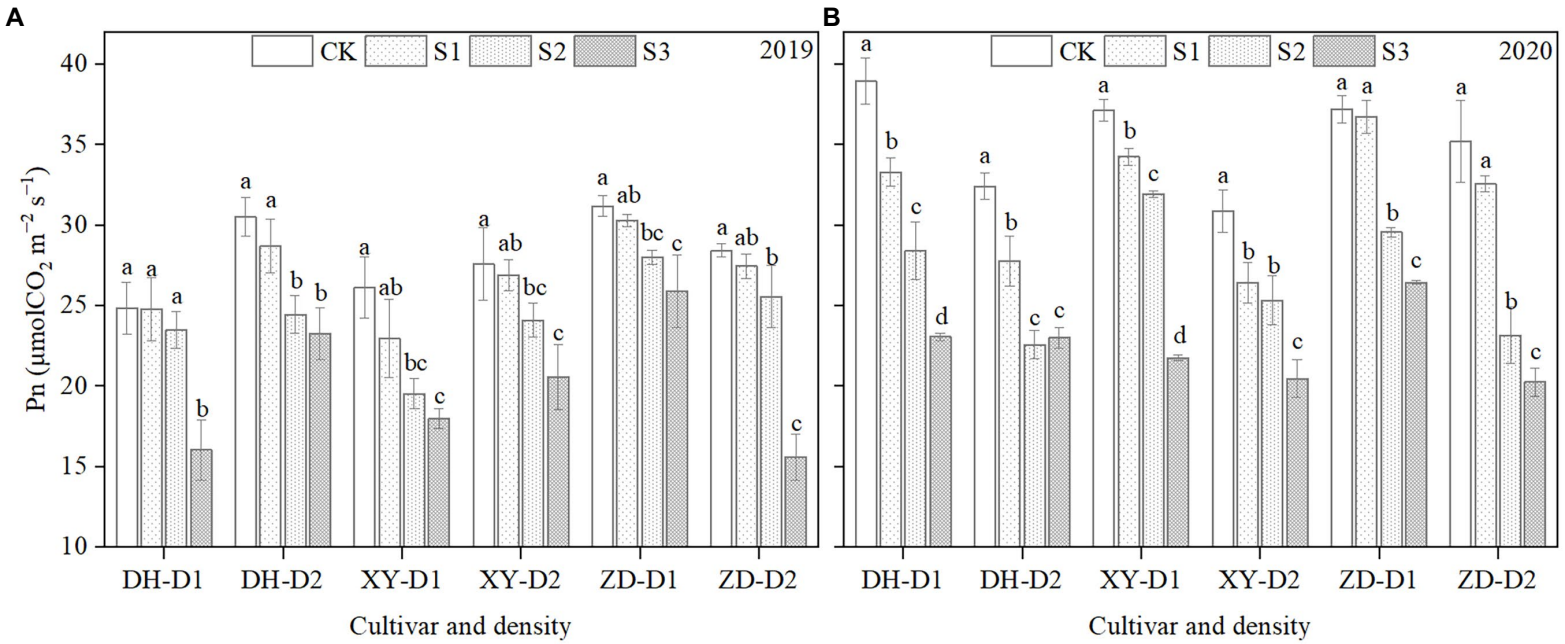
Ear leaf photosynthetic rate (*P*_n_) at the grain-filling stage for maize grown in 2019 **(A)** and 2020 **(B)**. DH, Denghai618; XY, Xianyu 335; ZD, Zhengdan 958; CK, natural light; S1, 85% of natural light (15% shaded); S2, 70% of natural light; S3, 50% of natural light; D1, 7.5 × 10^4^ plants ha^−1^; and D2, 12 × 10^4^ plants ha^−1^. Error bars represent the SD of three replicates. Lowercase letters indicate statistically significant differences (*p* < 0.05).

As shown in Figure 1, ear leaf photosynthetic rate (*P*_n_) decreased significantly in all three cultivars with shading in 2019 (Figure 1A) and 2020 (Figure 1B). On average across years, cultivars, and densities, the mean *P*_n_ decreased by 7.6, 17.6, and 32.6% in treatment groups S1, S2, and S3, respectively, compared with CK. On average across years, shading levels, and densities, mean *P*_n_ decreased in cultivars XY335, ZD958, and DH618 by 19.9, 18.6, and 17.5%, respectively, compared with CK. On average across years, shading levels, and cultivars, mean Pn decreased by 16.4 and 20.9% under the D1 and D2 planting densities, respectively, compared to CK.

### Quantitative Relationship Between Grain Filling Parameters and Solar Radiation

**Figure 2:**
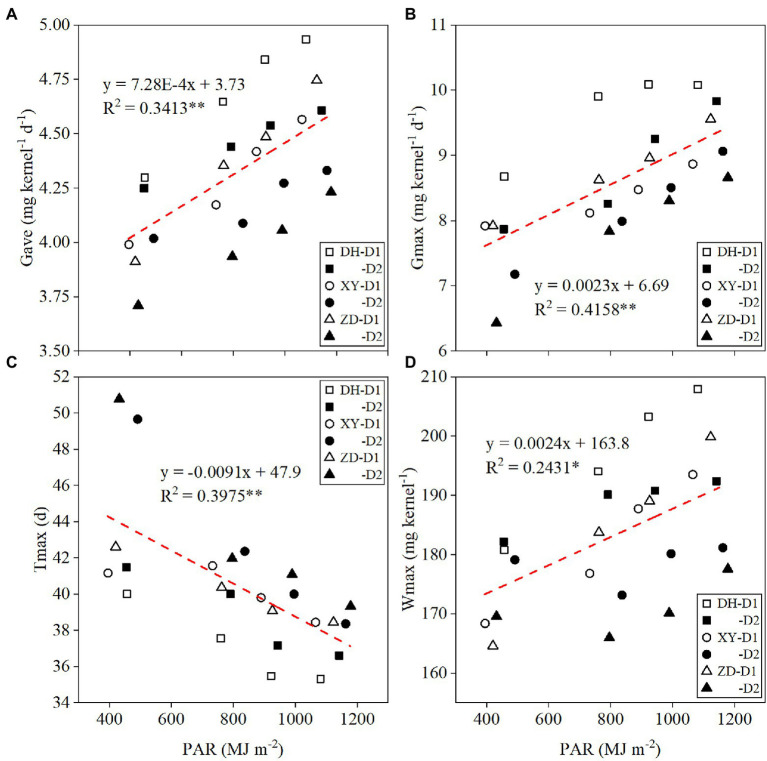
Relationships between maize grain filling parameters and [*G*_ave_, average grain filling rate **(A)**; *G*_max_, maximum grain-filling rate **(B)**; *T*_max_, time to maximum grain-filling rate **(C)**; *W*_max_, kernel weight at *T*_max_ **(D)**] photosynthetically active radiation (PAR) under different treatments. *G*_ave_, average grain filling rate; *G*_max_, maximum grain-filling rate; *T*_max_, time to maximum grain-filling rate; *W*_max_, kernel weight at *T*_max_; DH, Denghai618; XY, Xianyu 335; ZD, Zhengdan 958; D1, 7.5 × 10^4^ plants ha^−1^; and D2, 12 × 10^4^ plants ha^−1^. ^*^p < 0.05; ^**^p < 0.01.

**Figure 3:**
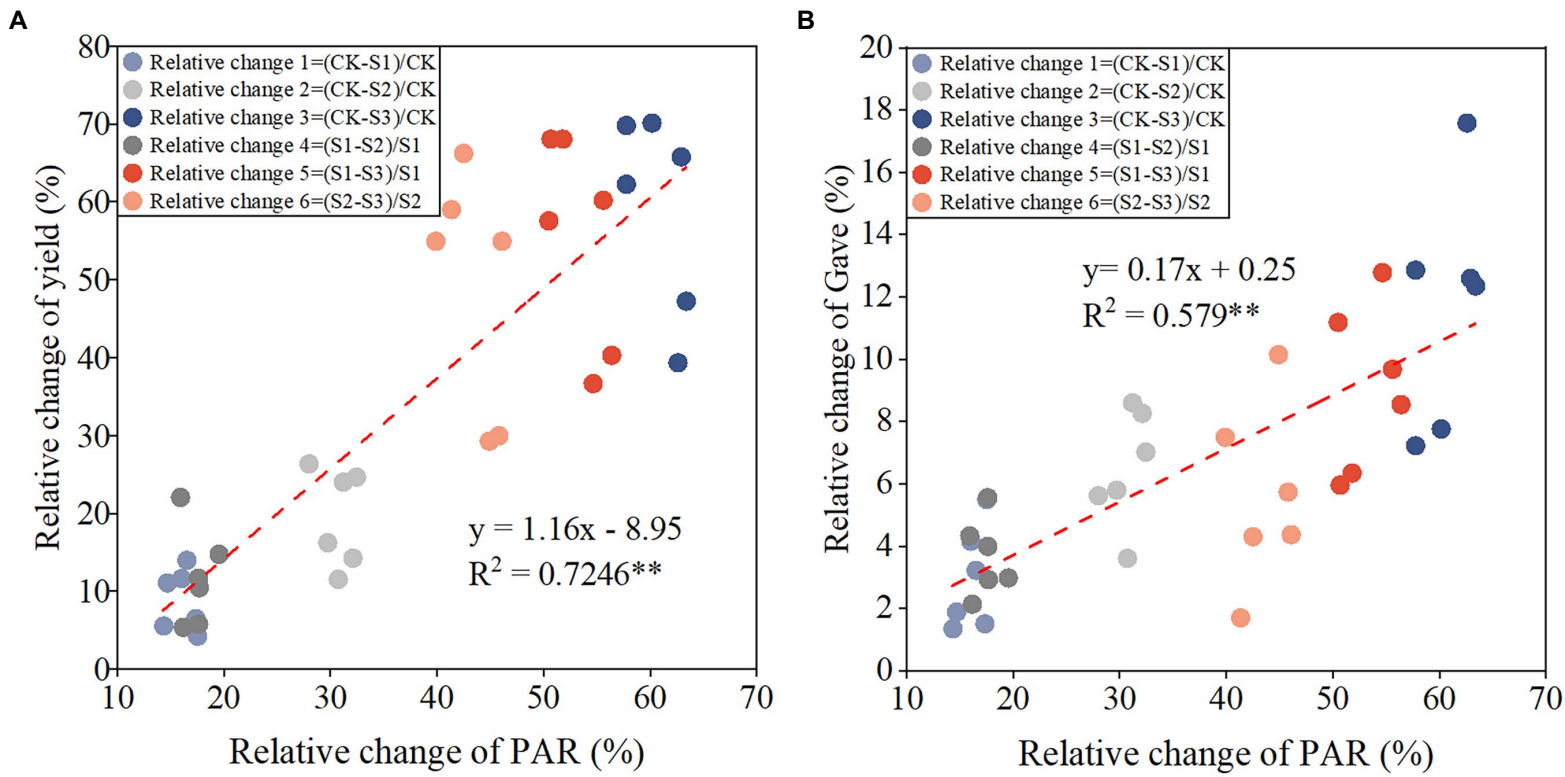
Relationships between relative change of PAR and relative changes of yield **(A)** and average grain filling rate (*G*_ave_; **B**). The equations for relative changes of PAR, yield, and *G*_ave_ across 2 years and planting densities are inset in the top left corner of each graph. ^**^p < 0.01.

*G*_ave_, *G*_max_, and *W*_max_ were significantly and positively correlated with PAR, whereas *T*_max_ was negatively correlated with PAR (Figure 2). With every 100 MJ m^−2^ increase in PAR, *G*_ave_ (Figure 2A), *G*_max_ (Figure 2B), and *W*_max_ (Figure 2D) increased by 0.073, 0.23 mg kernel^−1^ day^−1^, and 0.24 mg kernel^−1^, respectively, and *T*_max_ (Figure 2C) was delayed by 0.91 days. Relative changes in yield and *G*_ave_ between different shading treatments were significantly positively correlated with relative changes in PAR (Figure 3). With every 1% increase in PAR, yield (Figure 3A) and *G*_ave_ (Figure 3B) increased by 1.16 and 0.17%, respectively.

We found that *G*_ave_, *G*_max_, *W*_max_, and *P*_n_ were significantly positively correlated with grain weight and yield. Furthermore, *T*_max_ was delayed with increased shading levels and was significantly negatively correlated with grain weight and yield (Table 4). Significant interaction influences of year (Y), cultivar (C), planting densities (D), and shading level (S) on *y*_P_, *G*_ave_, *G*_max_, *T*_max_, and *W*_max_ existed (Table 5). As shown in Table 5, *y*_P_ and *W*_max_ were significantly affected by the interaction of Y × D. *y*_P_, *T*_max_, and *W*_max_ were significantly affected by the interactions of Y × C and Y × S. *y*_P_, *W*_max_, and *G*_max_ were significantly affected by the interactions of D × C and D × S. *T*_max_ was significantly affected by the interaction of D × C. *G*_ave_ was significantly affected by the interaction of Y × D × S. *y*_P_, *T*_max_, *W*_max_, and *G*_max_ were significantly affected by the interactions of Y × D × C, Y × D × S, Y × C × S, D × C × S, and Y × D × C × S.

**Table 4:** Correlation coefficients for yield, PAR, *P*_n_, *y*_p_, and grain filling parameters.

	PAR	G_ave_	Gmax	Tmax	Wmax	Pn	y_p_
G_ave_	0.621^**^						
Gmax	0.668^**^	0.923^**^					
Tmax	−0.653^**^	−0.837^**^	−0.927^**^				
Wmax	0.525^**^	0.957^**^	0.815^**^	−0.675^**^			
Pn	0.857^**^	0.673^**^	0.707^**^	−0.686^**^	0.552^**^		
*y*_p_	0.603^**^	0.994^**^	0.916^**^	−0.829^**^	0.970^**^	0.645^**^	
Yield	0.932^**^	0.614^**^	0.659^**^	−0.661^**^	0.513^*^	0.805^**^	0.593^**^

PAR, photosynthetically active radiation; *G*_ave_, average grain filling rate; *G*_max_, maximum grain-filling rate; *T*_max_, time to maximum grain filling rate; *W*_max_, kernel weight at *T*_max_; *P*_n_, ear leaf photosynthetic rate; and *y*_p_, potential grain weight (*x* = 80).

* *p < 0.05*;

** *p < 0.01*.

**Table 5:** Interaction influences of year (Y), cultivar (C), planting densities (D), and shading level (S) on grain weight [*y*_P_ (*x* = 80)], average grain filling rate (*G*_ave_), maximum grain-filling rate (*G*_max_), time to maximum grain-filling rate (*T*_max_), and the kernel weight at *T*_max_ (*W*_max_).

Source of variation	*y*_p_	*G*_ave_	*T*_max_	*W*_max_	*G*_max_
Y	**	*	**	**	**
D	ns	ns	**	ns	**
C	**	**	**	**	**
S	**	*	**	**	**
Y × D	*	ns	ns	*	ns
Y × C	**	ns	**	**	ns
Y × S	*	ns	**	*	ns
D × C	**	ns	**	**	**
D × S	**	ns	ns	**	**
C × S	**	ns	**	**	ns
Y × D × C	**	ns	**	**	**
Y × D × S	*	*	**	*	**
Y × C × S	**	ns	**	**	**
D × C × S	**	ns	**	**	**
Y × D × C × S	**	ns	**	**	**

* Significant differences at *p* < 0.05.

** Significant differences at *p* < 0.01.

ns represents no significant difference.

## Discussion

Maize yield formation is determined by grain filling capacity and final grain weight (Zhang et al., 2007b; Chen et al., 2014). Solar radiation is the energy source for photoassimilates (Shi et al., 2013), which are the ultimate driving factor of maize development and growth (Hou et al., 2021). Previous studies concluded that plant growth was more severely inhibited by low light as the level of shading increased (Bidhari et al., 2021). The present study supported those findings; we here found that shading negatively affected yield (Table 2), final grain weight, grain filling parameters (Table 3), and Pn (Figure 1), with higher levels of shade having greater effects. Other researchers have shown that the correlative coefficient of *G*_ave_ and *G*_max_ to 100-grain weight is highest in the late grain filling stage (Zhang et al., 2007a; Liu et al., 2013). Additionally, Zhang et al. (2007b) reported that this change could be a result of photosynthesis during grain filling. The results of the present study are consistent with that report, showing that *P*_n_ decreased significantly in all three cultivars in response to shading (Figure 1), which may be the underlying cause of the decrease in final grain weight. It has also previously been reported that decreasing both photosynthesis and the activity of key starch synthesis enzymes limits photosynthesis (Lu et al., 2013; Rivera-Amado et al., 2020; Feng et al., 2021) and caused decreases in transportation and distribution of photosynthates from leaves to grains (Zhang et al., 2008; Kromdijk et al., 2016; Gao et al., 2020; Feng et al., 2021). In addition, temperature condition during grain filling is one of the most important climatic drivers that determine the rate of crop development and biomass accumulation (Borrás et al., 2009; Kromdijk and Long, 2016; Hou et al., 2021). Previous studies have shown maize photosynthesis and grain filling rate are limited by temperature (Jia et al., 2018). However, there are many studies reported that there were no significant changes in the relative humidity and temperature at different positions inside and outside the shade shelter, after shading treatments (Zhang et al., 2007b; Feng et al., 2021). Similarly, in this study, a distance of 1.5 m was maintained between the top of the maize canopy and the shade nets to keep microclimate conditions consistent with the unshaded treatment. But for S3 treatment, the temperature might be decreased after shading which together with decreased light affected the growth of maize. This needs further research in the future.

A quantitative understanding of the factors driving changes in grain filling is essential to developing agricultural information technology, crop growth models, and crop options for adaptation to climate change (Hou et al., 2021; Liu et al., 2021a; Tao et al., 2022). There is a wide geographical range in which Chinese maize is grown; one of large differences between regions is the level of solar radiation (Liu et al., 2021a). Xinjiang is in Northwest China and has the most abundant light radiation (Xue et al., 2016; Yang et al., 2020). Shading treatments in this region can simulate the light environment in other regions and allow quantitative analysis of the relationship between maize growth and solar radiation. We here confirmed the quantitative relationships between maize grain filling parameters and PAR using different shading treatments and planting densities (Figure 2), which had not previously been widely reported (Chen et al., 2014). The study design allowed us to establish the relationship between relative changes in PAR and maize yield/*G*_ave_ (Figure 3). These data can be used to estimate the yield and *G*_ave_ of maize in different years or regions with varying levels of solar radiation, and contribute to the improvement of crop models.

Grain filling rate is affected by both genotype and environmental conditions, and light stress responses vary by genotype (Li et al., 2005). However, Rizzo et al. (2022) concluded that genetic improvement accounted for only 13% of yield increase, and that if genetic progress in yield potential was also slowing in other environments and crops, future crop-yield gains would increasingly rely on improved agronomic practices. Additionally, Li and Wang (2009) reported that breeding cultivars tolerant to high planting density and other adverse conditions would be the most effective cultivation measure for improving maize grain yield. Some studies also stated that as planting density increases the number of grains per ear decreases, and the maximum and average maize grain-filling rate significantly decrease (Jia et al., 2018). Previous studies also showed that the photosynthate produced under high density and weak light could not satisfy yield formation (Yang et al., 2021a). Besides, shading delayed vegetative and reproductive growth, and reduced the kernel number per ear and kernel weight because of the limited assimilates supply to the developing ear shoot (Cui et al., 2015; Yang et al., 2021b). In this study, shading had greater effects on *G*_ave_ and *W*_max_ at low planting density (D1), but smaller effects at high planting density (D2) which might be due to that *G*_ave_ and *W*_max_ under CK were much higher at low density than that at high density. In other words, under the higher planting density of D2, self-shading was more prominent by denser canopies and increased the proportion of partially shaded leaves (Kromdijk et al., 2016), therefore the shading treatments were less consequential. Moreover, the average yield and grain weight of DH618 plants were generally higher compared to the other two cultivars (Tables 2, 3). This may be because *G*_ave_ and other grain filling parameters were higher in cultivar DH618 than the other two cultivars after shading (Table 3). Therefore, we think that cultivars DH618 was a kind of suitable for dense planting under insufficient light conditions (Table 2). In addition, the decreases in grain filling parameters were smaller in DH618 than in XY335 and ZD958 after shading. This could be because cultivar DH618 is an erect-type hybrid, which can intercept as much solar radiation as possible (Liu et al., 2021a).

## Conclusion

Understanding the quantitative relationship between solar radiation and grain filling parameters of maize is essential for improving maize production and developing options for adaptation to climate change. In this study, it was found that there were significant differences in maize final grain weight and other grain filling parameters in response to shading treatments. Quantitative relationships were discovered between PAR and grain yield, *G*_ave_, *G*_max_, *T*_max_, and *W*_max_. DH618 had better grain filling ability and tolerance to high planting density than the other two cultivars under weak light conditions. This type of cultivar should be selected and bred for low light adaptation to achieve high grain yield in the face of climate change, particularly the decrease in solar radiation.

## Data Availability Statement

The original contributions presented in the study are included in the article/supplementary material, further inquiries can be directed to the corresponding authors.

## Author Contributions

YY carried out the measurements and data analysis and wrote the manuscript. YY, PH, and SL designed the experiment. YY, GL, XG, and WL performed the study. YY, GL, XG, WL, JX, BM, RX, KW, PH, and SL made substantial contributions to conception and critically revised the manuscript. All authors contributed to the article and approved the submitted version.

## Funding

This study was supported by the National Natural Science Foundation of China (31871558 and 32172118), the National Key Research and Development Program of China (2016YFD0300110 and 2016YFD0300101), and the China Agriculture Research System of MOF and MARA.

## Conflict of Interest

The authors declare that the research was conducted in the absence of any commercial or financial relationships that could be construed as a potential conflict of interest.

## Publisher’s Note

All claims expressed in this article are solely those of the authors and do not necessarily represent those of their affiliated organizations, or those of the publisher, the editors and the reviewers. Any product that may be evaluated in this article, or claim that may be made by its manufacturer, is not guaranteed or endorsed by the publisher.
